# Runtime-Robust Edge Inference System with Masking-Based Partial Update on Dynamic Reconfigurable FPGA

**DOI:** 10.3390/s25247448

**Published:** 2025-12-07

**Authors:** Myeongjin Kang, Daejin Park

**Affiliations:** 1 School of Electronic and Electrical Engineering, Kyungpook National University, Daegu 41566, Republic of Korea; 2 School of Electronics Engineering, Kyungpook National University, Daegu 41566, Republic of Korea

**Keywords:** edge-cloud system, FPGA accelerator, learning accelerator, dynamic partial reconfiguration

## Abstract

Edge inference systems must sustain real-time performance under dynamic environments such as sensor noise, illumination change, and new object classes. Conventional edge devices deploy static offline-trained models, causing accuracy degradation when the input distribution drifts. This study proposes a runtime-robust edge inference framework that enables continuous adaptation without interrupting execution. The edge device partitions its memory into active and adaptive regions, applying task-specific masked updates generated by a server-side FPGA. The FPGA performs layer-wise importance analysis, partial retraining, and adaptive mask generation using dynamic partial reconfiguration (DPR) to minimize reconfiguration delay. Experiments on MNIST, CIFAR-10, and Tiny ImageNet show that the proposed method reduces adaptation latency by up to 1.3× compared with GPU full retraining while cutting the communication cost to 28% of full model transmission. These results demonstrate that combining masking-based selective updates with FPGA DPR acceleration achieves real-time adaptability, low latency, and communication-efficient learning in cloud–edge collaborative environments.

## 1. Introduction

Edge inference must deliver real-time responses under changing conditions such as sensor noise, lighting shifts, and the appearance of previously unseen classes. Many edge devices execute offline-trained models stored in on-chip flash and keep the deployed structure fixed during operation. This static deployment limits adaptation and causes accuracy degradation once the input distribution drifts [1,2]. In practical edge deployments, sensor data often undergoes continuous variation due to environmental dynamics such as illumination change, motion blur, temperature drift, or sensor degradation. These variations introduce a distribution shift between the training data and the live sensor input, causing inference accuracy to degrade over time. To ensure robustness under such conditions, the edge device must adapt its model parameters in accordance with sensor-induced feature changes.

**Figure 1:**
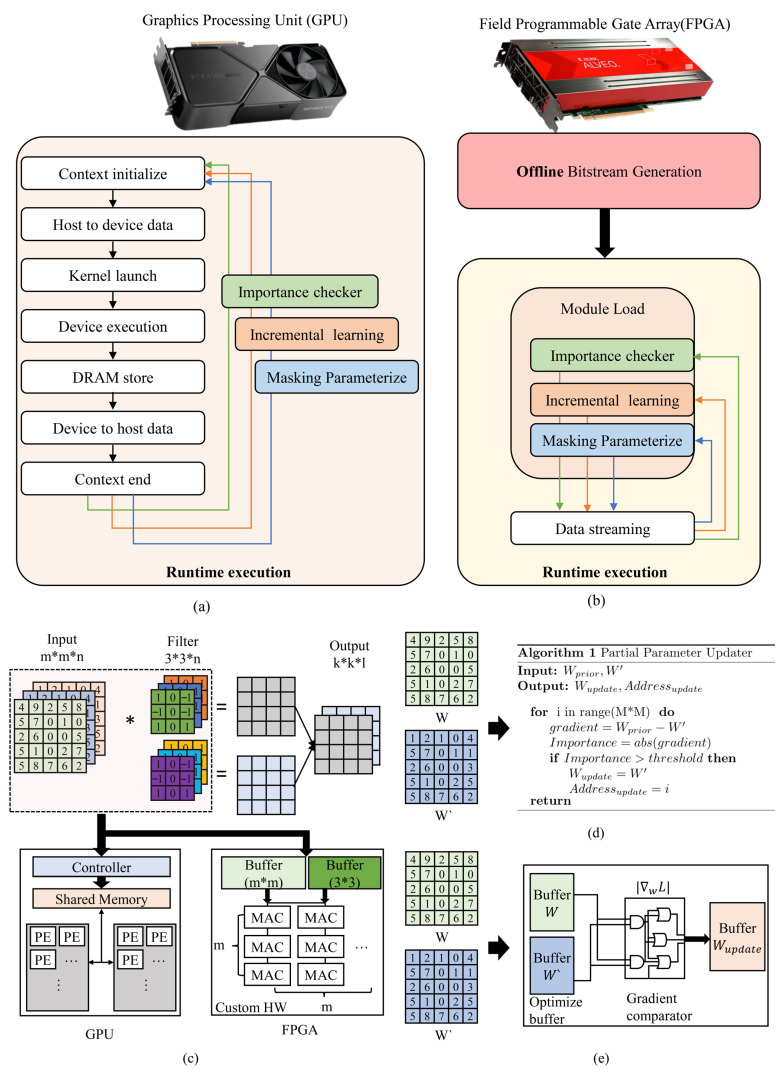
Comparison of GPU-based sequential runtime and FPGA-based DPR pipeline. (**a**) Conventional GPU runtime flow repeatedly accelerates context initialization, kernel reloads, and data transfers. (**b**) A DPR FPGA that repeatedly performs accelerates various operations through module loading. (**c**) The difference between GPUs, which have fixed bandwidth and shared memory, and FPGAs, which can customize MAC operators, buffers, etc. (**d**) GPU MAC operations through the compiler and scheduler based on the algorithm. (**e**) Hardware-based FPGA MAC operations without a processor.

Although continuous retraining could restore performance, full-model retraining on edge devices is infeasible due to limited computational capability, memory capacity, and power constraints [3]. Furthermore, transmitting an entire model from the cloud during every update causes substantial communication overhead and latency, which becomes impractical for time-sensitive or bandwidth-limited edge deployments. As shown in Figure 1, conventional cloud–edge pipelines typically rely on batch learning and static redeployment cycles. These approaches interrupt ongoing inference and fail to react promptly to sudden distribution shifts. Moreover, GPU-based acceleration suffers from architectural rigidity: kernels must be frequently reloaded, memory must be re-synchronized, and contexts must be repeatedly switched across learning stages [4,5,6]. Such overhead prevents GPUs from supporting fine-grained, layer-wise updates at runtime, making real-time adaptation impractical.

To overcome these limitations, this paper proposes a runtime-robust edge inference architecture that enables dynamic model updates without interrupting ongoing inference. The edge device employs a memory-separated structure that maintains an active model region and an adaptive region. Only masked weight deltas corresponding to highly influential parameters are transmitted from the server, significantly reducing communication cost compared with full-model updates. After receiving an update, the edge activates new parameters through a fast pointer swap rather than reloading the entire model, thereby eliminating inference downtime. This selective and incremental update mechanism allows the system to adapt rapidly to new classes, noise-induced feature drift, and other dynamic conditions, achieving real-time responsiveness unattainable with prior cloud–edge or GPU-based frameworks.

Overall, the proposed architecture forms a unified cloud–edge adaptive learning framework that combines masking-based updates, FPGA reconfigurability, and efficient communication. It provides runtime adaptability under dynamic environments while maintaining service continuity and reducing overhead in both computation and data transfer [7,8].

The main contributions of this paper are as follows. First, a runtime adaptive edge inference architecture is proposed that flexibly replaces model weights during execution without interrupting inference. Second, a server-side retraining pipeline accelerated by a PCIe-connected FPGA performs on-demand incremental learning and selective parameter optimization in response to runtime events. Third, dynamic partial reconfiguration (DPR) is utilized to enable fast layer-wise reconfiguration [9,10,11], providing scenario-specific acceleration without full FPGA reprogramming. Fourth, a masking-based selective weight update method is designed to transmit only the most influential parameters, effectively reducing update payloads. Fifth, an event-driven scheduling mechanism overlaps server retraining and edge inference to maintain continuous operation. Finally, a communication-efficient update path employing sparse and quantized transmission minimizes bandwidth usage while preserving real-time responsiveness.

The remainder of this paper is organized as follows. Section 2 reviews related work on selective retraining, FPGA-based acceleration, and communication-efficient updates. Section 3 describes the proposed architecture and the partial reconfiguration workflow. Section 4 presents experimental results on learning speed, communication efficiency, and runtime robustness. Section 5 concludes the paper and discusses potential future extensions toward adaptive edge–cloud learning systems.

## 2. Related Work

### 2.1. Retraining Layer Selection via Importance Checker

Selective retraining methods identify layers or parameters that most affect loss under distribution shift [12,13]. A first-order criterion evaluates the magnitude of the gradient of the loss L with respect to each parameter $w_{i}$:

(1) $$s_{i}^{\mathrm{grad}}=\left|\frac{\partial L}{\partial w_{i}}\right|,$$

where $\frac{\partial L}{\partial w_{i}}$ denotes the instantaneous gradient and $s_{i}^{\mathrm{grad}}$ represents the local sensitivity of parameter $w_{i}$.

A Taylor-series-based approximation additionally incorporates the parameter magnitude. Using a first-order expansion,

(2) $$\Delta L\approx \sum_{i}\frac{\partial L}{\partial w_{i}}\;\Delta w_{i}\;\Rightarrow \;s_{i}^{\mathrm{taylor}}=\left|w_{i}\frac{\partial L}{\partial w_{i}}\right|,$$

where $\Delta w_{i}$ denotes a hypothetical perturbation. The score $s_{i}^{\mathrm{taylor}}$ captures the combined effect of the gradient and parameter magnitude and is widely used in pruning and selective retraining.

Second-order methods approximate curvature by using the diagonal Fisher information:

(3) $$F_{i}\approx E\left[{\left(\frac{\partial \log p_{\theta }\left(y|x\right)}{\partial w_{i}}\right)}^{2}\right],$$

where $p_{\theta }\left(y|x\right)$ is the model likelihood. Using $F_{i}$, two importance formulations are commonly used:

(4) $$s_{i}^{\mathrm{fisher}}=F_{i}\;\Delta w_{i}^{2},\;s_{i}^{\mathrm{ewc}}=F_{i}\;{\left(w_{i}-w_{i}^{⋆}\right)}^{2},$$

where $w_{i}^{⋆}$ denotes the previously consolidated parameter value, as in elastic weight consolidation.

At the layer level, parameter scores are aggregated to obtain a layer importance value:

(5) $$S_{\ell }=\frac{1}{|W_{\ell }|}\sum_{i\in W_{\ell }}s_{i},$$

where $W_{\ell }$ denotes the set of parameters in layer *ℓ*. Layers with the highest $S_{\ell }$ values are selected for retraining. Practical approximations include Hutchinson-based Hessian trace estimation, gradient-norm heatmaps for block-level selection, and classifier-only updates for new-class scenarios [14,15]. These techniques localize computation to the most influential portions of the network and significantly reduce the communication and retraining cost under runtime adaptation.

### 2.2. FPGA Partial Replacement for Runtime Adaptation

Partial reconfiguration (PR) allows a static shell to remain active while a reconfigurable partition (RP) swaps hardware modules at runtime [16,17]. Common practice fixes the RP boundary with standardized interfaces such as AXI4 Lite for control and AXI4 Stream or memory-mapped AXI for data, with clock and reset isolation to ensure bitstream compatibility across reconfigurable modules. Before reconfiguration, in-flight transactions are quiesced and drained to preserve correctness.

**Figure 2:**
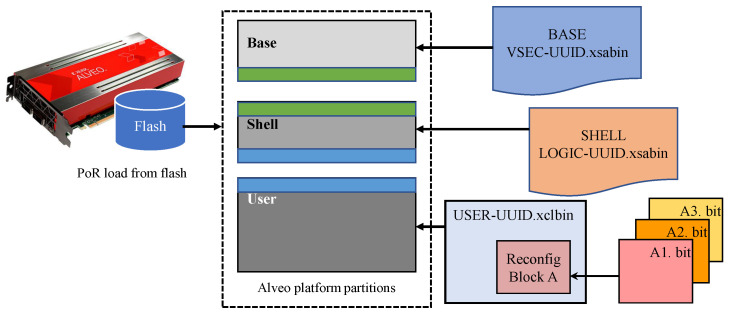
Alveo dynamic partial reconfiguration platform.

On data center FPGAs such as AMD Xilinx Alveo, as seen in Figure 2, prior systems use PR to switch convolution variants, precision-tuned kernels, or task-specific accelerators without full device reprogramming. HLS-assisted PR and incremental compilation toolflows enable catalogs of pre-built reconfigurable modules that can be selected at runtime [18,19]. Reported module swap latencies scale with the partial bitstream size and the configuration path, typically in the sub-second to low-second range. These hardware hot swaps complement software-only methods by enabling structural changes in the accelerator pipeline under latency and power constraints [20].

### 2.3. GPU-FPGA Comparison for Runtime Adaptation and Update

As seen in Figure 1, GPU pipelines excel at dense, throughput-oriented computation and can apply updates by either swapping weights in an existing engine or by rebuilding and reloading an engine when the network structure, precision, or sparsity configuration changes.

The former path minimizes downtime but restricts adaptation to fixed architectures [21]. The latter path enables structural changes but introduces rebuild and load costs that can rise with model size and optimization depth [22].

FPGA PR offers a complementary trade-off. A pre-built catalog of reconfigurable modules supports structural adaptation through runtime module swaps, while the static shell continues to serve I/O and memory. Let $T_{\mathrm{apply}}^{\mathrm{GPU}}$ and $T_{\mathrm{apply}}^{\mathrm{FPGA}}$ denote the application phase of an update:

(6) $$T_{\mathrm{apply}}^{\mathrm{GPU}}=\left\{\begin{array}{cc} T_{\mathrm{engine\_build}} & \mathrm{weights}\;\mathrm{only}\;\mathrm{swap}\\ T_{\mathrm{engine\_build}}+T_{\mathrm{engine\_build}} & \mathrm{engine}\;\mathrm{rebuild} \end{array}\right.,\;T_{\mathrm{apply}}^{\mathrm{FPGA}}=T_{\mathrm{PR\_load}}+T_{\mathrm{warmup}}.$$

In practice, weight-only swaps on GPUs achieve very low latency for fixed architecture adaptation. When structural changes are required, PR-based swaps can avoid full device reprogramming and offer sub-second to low-second update times depending on partial bitstream size while keeping the static shell active. Comparative studies therefore evaluate end-to-end update latency, tail latency impact during the update window, and energy per update, as well as accuracy gain per transmitted megabyte in end-to-end cloud–edge scenarios [23].

### 2.4. Compressed Partial Update and Masking

Communication-efficient adaptation transmits only the most useful parameters. Masking-based partial updates form an importance mask M and send only the corresponding deltas $\Delta w_{M}$:

(7) $$M=\{i\;:\;s_{i}\geq \tau \;\mathrm{or}\;\mathrm{top}\text{-}k\},\;\Delta w_{M}=\left\{\Delta w_{i}|i\in M\right\},$$

with policies based on $\left|\Delta w_{i}\right|$, gradient magnitude, Fisher-weighted scores, or layer-wise constraints. Payload is further reduced by quantization to eight- or four-bit representations and by sparse encodings such as CSR or coordinate lists. Related streams include gradient sparsification with error feedback, sign-based updates, and parameter efficient finetuning that acts as an implicit mask through low rank or adapter paths [24,25].

For cloud–edge systems, masked updates can be applied in a shadow buffer and activated via an atomic pointer swap after integrity checks, thus preserving online inference [26,27]. Compared with full model transfers, compressed partial updates improve accuracy gain per transmitted megabyte and reduce downtime, which aligns with runtime robustness requirements under dynamic inputs.

## 3. Proposed Architecture

This section presents a runtime adaptive edge inference architecture that combines device-side memory separation with masking-based partial updates and a server-side pipeline accelerated by a PCIe-connected dynamic reconfigurable FPGA. The design targets low-latency adaptation under dynamic inputs without interrupting device inference. The system integrates three pillars that operate concurrently. The server detects scenarios and learns selective changes. The FPGA accelerates importance scoring and layer-wise retraining and supports scenario-specific acceleration through partial reconfiguration. The edge applies masked updates in a shadow region and activates them through an atomic pointer swap.

### 3.1. System Overview

**Figure 3:**
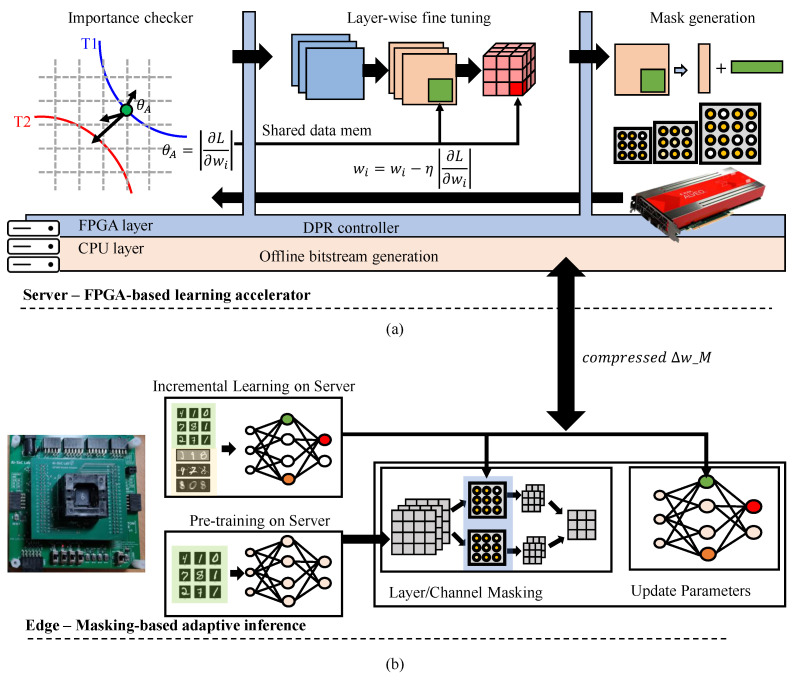
Overall edge-cloud collaborative architecture for runtime adaptive inference. (**a**) A server-side FPGA learning accelerator leveraging layer-by-layer fine-tuning, importance scoring, and mask generation based on a DPR controller. (**b**) Edge side incremental adaptive inference based on masking and real-time learning weight transfer.

Figure 3 illustrates the overall runtime-adaptive inference architecture. The system consists of two cooperative domains: the edge device performing incremental inference and the server-side FPGA accelerator responsible for adaptive retraining and mask generation. Both operate concurrently to maintain inference continuity and adaptivity under dynamically changing input conditions.

**Figure 4:**
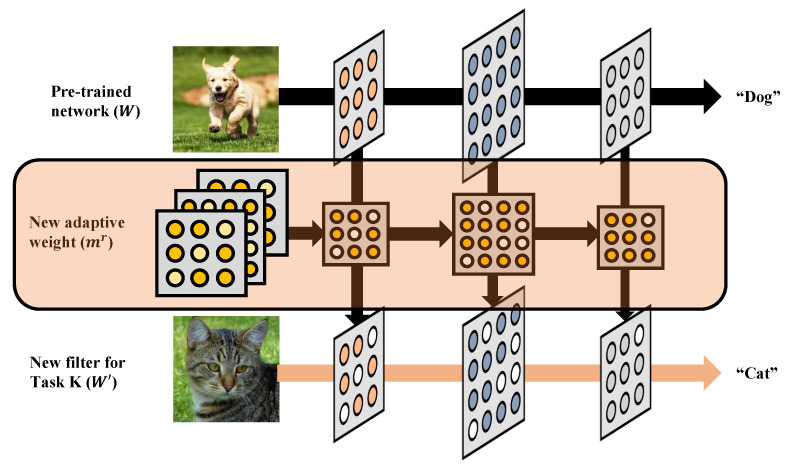
Task-adaptive partial update method.

On the edge side, the system divides memory into two functional regions rather than maintaining a single shadow buffer. The active memory stores the baseline model and parameters frequently used for inference, while the adaptive memory holds task-specific masks and selectively updated parameters provided by the server. Each mask $M_{k}$ corresponds to a particular environmental or contextual condition. As seen in Figure 4, during runtime, the edge retrieves the corresponding mask and reconstructs a new effective weight by combining stored weights and masked updates:

(8) $$W_{\mathrm{eff}}=W_{\mathrm{base}}+\sum_{k=1}^{K}M_{k}⊙\Delta W_{k}$$

where ⊙ denotes element-wise selection. Although this requires lightweight additional computation, it eliminates the need for full weight replacement and supports incremental inference [28] that dynamically adapts to the current context. As a result, inference continues seamlessly while parameter adaptation occurs in parallel.

The server receives statistical summaries and inference results from the edge device, analyzes performance degradation, and identifies the cause of change. Based on the detected scenario, the server performs layer-wise retraining on the FPGA and generates a new mask Mnew together with its corresponding parameter deltas ΔWnew. These updates are compressed and transmitted to the edge for integration into the adaptive memory. Through this continuous collaboration, the edge maintains up-to-date model behavior without halting inference or requiring full model transmission.

This memory-partitioned structure provides several advantages. First, it enables runtime weight adaptation without model reloading, reducing latency and communication cost. Second, it allows task- or scenario-specific inference using previously stored masks, ensuring rapid recovery when similar situations recur. Finally, it provides a foundation for incremental learning and inference fusion, where partial updates and continuous inference coexist within a unified runtime pipeline.

### 3.2. Edge Runtime Mechanism with Mask-Based Adaptive Inference

The edge device performs inference continuously while adapting its parameters according to the current task or environment. Instead of performing full weight replacement, the edge selectively applies mask-based parameter fusion using pre-stored update information. Each task-specific mask encodes the indices and magnitudes of parameters that are most sensitive to the corresponding scenario, enabling incremental inference without reloading the entire model.

To support runtime adaptation, the edge memory is divided into two logical regions: the base memory that holds the static model $W_{base}$ and the adaptive memory that stores multiple sets of task-specific parameter deltas Wk and their corresponding masks Mk. When the input context or task label indicates a change, the edge retrieves the relevant mask and constructs a new effective weight tensor:

(9) $$W_{\mathrm{eff}}^{\left(t\right)}=W_{\mathrm{base}}+M_{\mathrm{sel}(t)}⊙\Delta W_{\mathrm{sel}(t)}$$

where ⊙ denotes element-wise multiplication between the selected mask and its associated parameter delta. This operation adaptively modifies only the parameters relevant to the detected task or environmental condition, generating a lightweight and task-optimized weight configuration. Since the computation involves sparse element-wise updates instead of full model replacement, it requires minimal overhead and remains feasible even for microcontroller-level edge devices.

Each mask $M_{k}$ is designed by the server-side FPGA through layer-wise importance analysis, and it selectively activates a subset of parameters that significantly influence accuracy for a given condition. By maintaining multiple masks within the adaptive memory, the edge device can dynamically switch between different scenarios or tasks in real time. This mechanism enables task-aware runtime inference, allowing parameter adaptation to occur within a single inference cycle without requiring communication with the server.

The edge runtime engine follows a lightweight policy module that decides which mask to apply. This policy can rely on metadata from the sensor stream or previously observed inference degradation. If the system detects a new or unseen scenario for which no mask exists, the edge flags a retraining request to the server, which then produces a new $M_{\mathrm{new}}$ and $W_{new}$ pair through FPGA-accelerated incremental learning. The update is transmitted back and stored in the adaptive memory for future use.

This design provides three key benefits. First, it allows continuous inference without interrupting execution, as updates are integrated locally through simple weight fusion. Second, it supports task-level reuse of previously learned parameter deltas, reducing communication frequency with the server. Third, it achieves runtime adaptability where inference, parameter selection, and update coexist seamlessly on the edge device.

### 3.3. Server Runtime with FPGA DPR

**Figure 5:**
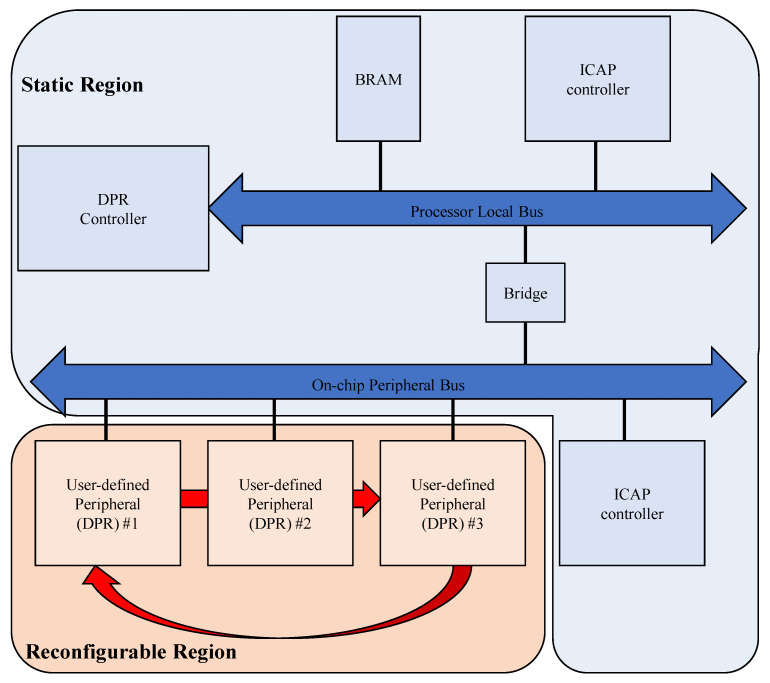
FPGA user-defined partial reconfigurable parts.

The server operates as an adaptive retraining platform that dynamically reconfigures FPGA hardware modules at runtime under the supervision of a CPU-based DPR controller. The FPGA is divided into a static region and an RP, as seen in Figure 5. The static region maintains PCIe and DDR interfaces, AXI interconnects, DMA engines, and control registers, ensuring that communication with both the host and the edge device remains uninterrupted during partial reconfiguration. The reconfigurable region hosts interchangeable acceleration modules for importance analysis, weight update, and adaptive mask generation. Each module follows a standardized interface based on AXI4 Lite for control and AXI4 Stream for data exchange, enabling module swapping without resynthesis or interface modification.

The CPU acts as a DPR controller, monitoring edge-side events and scheduling FPGA reconfiguration according to adaptation requirements. During normal operation, the importance analyzer module remains active, continuously evaluating sensitivity scores from recent inference data to identify parameters with a high impact on prediction accuracy. When an update request is triggered by the edge or when accumulated importance scores exceed a threshold, the CPU initiates partial reconfiguration to replace the current module with a weight update engine. After retraining and parameter generation, the CPU reconfigures the FPGA again to load the mask generator module, which extracts sparse and quantized parameter subsets for transmission to the edge. Once the update is completed, the FPGA reverts to the importance analyzer to resume runtime monitoring.

#### 3.3.1. Importance Analyzer

The importance analyzer identifies parameters and layers that most significantly influence the model’s output under the current data distribution. This module implements a hardware-accelerated approximation of the local gradient to estimate feature sensitivity in real time. Instead of executing full backpropagation, which requires global memory for intermediate activations and large-scale tensor operations, the FPGA computes a partial gradient using feature and error signals from the selected layer.

For an input feature vector $x_{L}$ and its output error signal $\delta_{L}=\partial L/\partial y_{L}$, the local gradient and importance score of parameter $w_{i}$ are computed as

(10) $$g_{i}=\delta_{i}\times x_{i},\;s_{i}=\left|g_{i}\right|,$$

where $s_{i}$ represents the magnitude of sensitivity for each parameter. This lightweight operation captures how strongly each weight contributes to loss variation without requiring a full backward pass. The computation is fully pipelined and parallelized across multiple DSP arrays, allowing element-wise operations to be performed every clock cycle.

#### 3.3.2. Partial Weight Update

The partial weight update engine performs selective retraining of model parameters based on the importance information obtained from the previous stage. Rather than updating the entire model as in conventional GPU-based training, this module focuses only on parameters whose importance scores exceed a predefined threshold. This approach reduces the computational load and memory bandwidth while maintaining comparable accuracy recovery.

Let $w^{\left(t\right)}$ denote the current weight vector at iteration *t*, and let $g=\delta_{L}⊙x_{L}$ represent the local gradient estimated by the importance analyzer. The updated parameter vector $w^{\left(t+1\right)}$ is computed as

(11) $$w_{i}^{\left(t+1\right)}=\left\{\begin{array}{cc} w_{i}^{\left(t\right)}-\eta g_{i} & s_{i}>\tau \\ w_{i}^{\left(t\right)} & \mathrm{otherwise} \end{array}\right.$$

where η is the learning rate, $s_{i}=\left|g_{i}\right|$ is the importance score, and τ is the threshold defined by the update policy. Only a small fraction of parameters satisfying $s_{i}>\tau$ are modified, thereby enabling rapid incremental learning and low latency adaptation.

#### 3.3.3. Adaptive Mask Generator

The adaptive mask generator constructs a selective update mask that determines which parameters are to be transmitted and updated during runtime. While the importance analyzer provides element-wise sensitivity values, the mask generator aggregates these scores under multiple policies depending on task objectives, resource availability, and environmental context. This stage thus acts as the bridge between importance estimation and parameter updating.

Given the importance scores $s=s_{1},s_{2},\ldots ,s_{N}$, the generator constructs a binary mask M according to a configurable policy function Φ(·):

(12) $$M_{i}=\left\{\begin{array}{cc} 1, & \mathrm{if}\;s_{i}\geq \tau \;\left(\mathrm{threshold}\;\mathrm{policy}\right),\\ 1, & \mathrm{if}\;i\in \mathrm{Top}\text{-}k(s)\;(\mathrm{Top}\text{-}k\;\mathrm{policy}),\\ 0, & \mathrm{otherwise}. \end{array}\right.$$

where τ denotes an importance threshold and *k* specifies the number of highest score elements to retain. The policy Φ is dynamically selected by the CPU controller according to the active update mode. Each policy yields a different sparsity ratio, enabling the system to balance adaptation quality against communication and power cost.

## 4. Experiments

### 4.1. Experimental Setup

**Figure 6:**
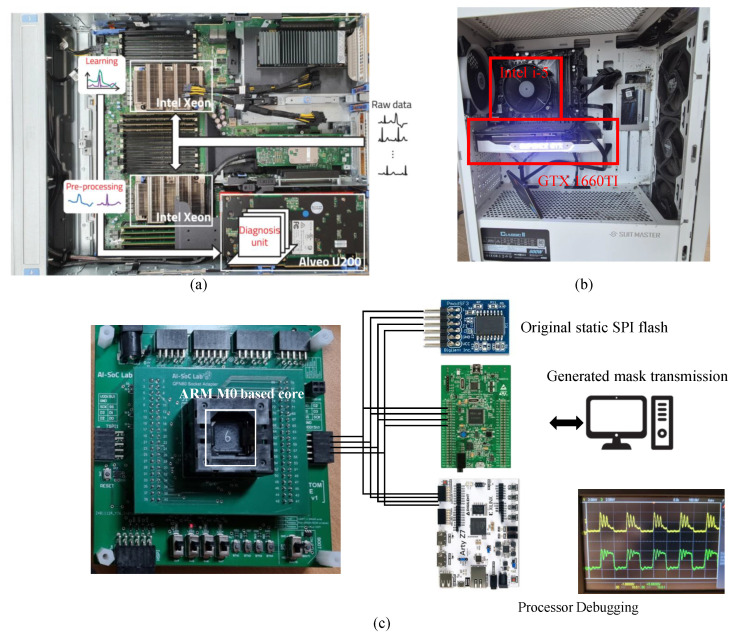
Experimental setup. (**a**) Server-side Xilinx Alveo U200 accelerator. (**b**) Server-side GPU accelerator. (**c**) Edge-side Arm core-based inference processor.

The proposed architecture was evaluated on both GPU- and FPGA-based platforms to compare the runtime adaptability and update efficiency as seen in Figure 6. For the GPU baseline, experiments were performed on a workstation equipped with an Intel Core i5-12400F CPU and an GTX 1660Ti GPU, Nvidia, Santa Clear, CA, USA, using PyTorch 2.0 for training and inference. The FPGA implementation was deployed on a Alveo U200 accelerator card, Xilinx, San Jose, CA, USA hosted in a dual CPU server system consisting of two Intel Xeon Bronze 3204 processors, Santa Clara, CA, USA (1.9 GHz, 6 cores each). All FPGA kernels were synthesized using the Xilinx Vitis 2023.1 toolchain with Vivado partial reconfiguration support. The host software managing reconfiguration and communication was implemented in C++ using XRT APIs. The edge-side inference processor is implemented on a custom ARM Cortex-M0-based microcontroller, Cambridge, UK operating at 48 MHz. The processor includes 128 KB of on-chip SRAM and 24 KB of DRAM, providing a highly resource-constrained environment representative of low-power edge platforms. During runtime, the processor loads the active weight region into SRAM, performs fixed-point convolution and fully connected operations, and applies masked parameter updates generated by the FPGA server. In addition, the Cortex-M0 periodically reports lightweight statistics—including prediction confidence, anomaly indicators, and feature drift metrics—to trigger server-side incremental retraining.

For performance evaluation, the experiments were conducted using the MNIST and CIFAR-10 datasets for incremental learning scenarios and a subset of Tiny ImageNet for noise adaptation tests. Each experiment consisted of three stages: importance estimation, partial update, and masked parameter transmission. The FPGA-based implementation was compared with the GPU full retraining baseline in terms of total adaptation latency, communication overhead, and accuracy recovery. All accuracy values were measured as the mean of five repeated runs.

### 4.2. Latency and Throughput Evaluation

**Figure 7:**
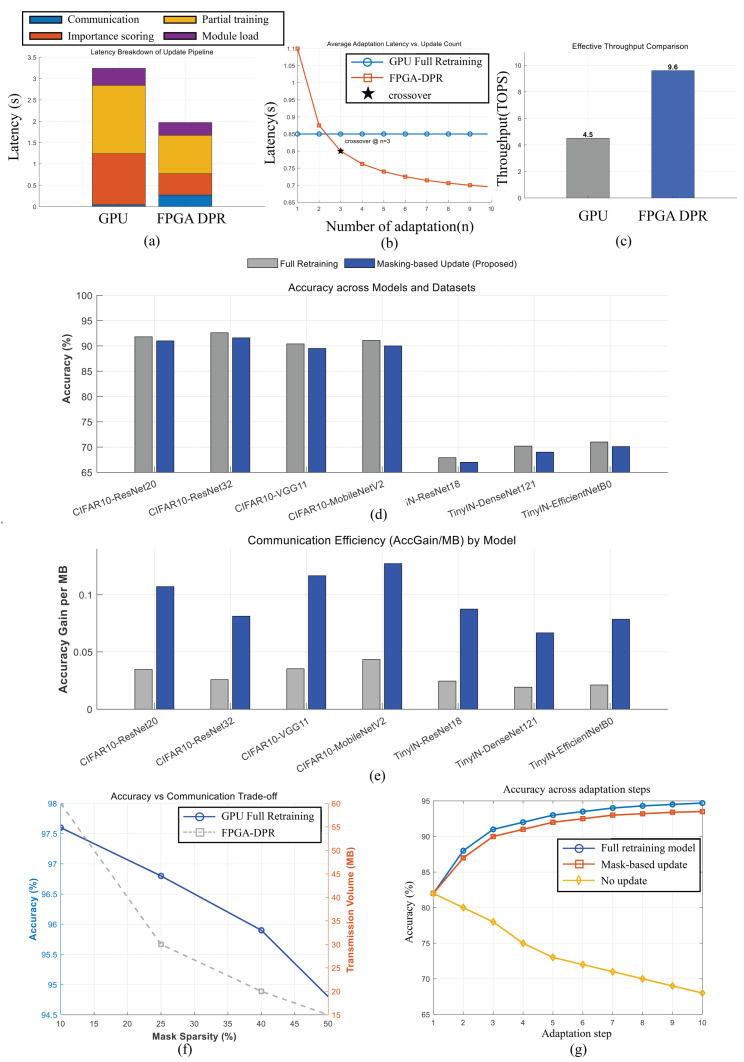
Experimental result. (**a**) GPU vs. FPGA latency. Sum of 1-time communication, importance scoring, and partial training PR load. (**b**) GPU vs. FPGA latency changes according to the number of adaptations. (**c**) GPU vs. FPGA total operation throughput. (**d**) Accuracy of full retraining and masking-based update across multiple datasets and models. (**e**) Communication efficiency of retraining and masking-based update across multiple datasets and models. (**f**) Accuracy versus communication trade-off in GPU and FPGA. (**g**) Accuracy comparison of the full retraining model, the mask-based update model, and no update across adaptation steps.

Figure 7 presents the latency comparison between the proposed FPGA-based adaptive update system and the GPU full retraining baseline. During the first adaptation cycle, the GPU achieved lower latency (0.78 s) than the FPGA (1.10 s), primarily because of the initial overhead associated with dynamic partial reconfiguration (DPR) and module loading. However, as adaptation events were repeated, the FPGA’s latency rapidly decreased and stabilized around 0.65 s, while the GPU’s retraining time remained nearly constant. After the third update iteration, the average adaptation latency of the FPGA dropped below that of the GPU, achieving up to a 1.3× overall speed advantage in steady-state operation.

The measured DPR latency ranged from 0.11 s to 0.17 s depending on the reconfigurable module size, occupying less than 20% of the total adaptation time after steady-state convergence. The FPGA kernel operated at 300 MHz, processing 32 feature elements per clock with an initiation interval of one, yielding an effective throughput of 9.6 TOPS for FP16 operations approximately 2.1× higher than the GPU baseline. Furthermore, the event-driven scheduling framework overlapped importance analysis, DPR execution, and data transmission, resulting in near-continuous operation with minimal idle time. Overall, the FPGA-DPR system demonstrated increasing efficiency with repeated updates, confirming its suitability for long-running adaptive inference pipelines where cumulative update cost dominates.

### 4.3. Accuracy Evaluation

In the accuracy evaluation across adaptation steps, the proposed system was tested under a progressively deteriorating noise environment, where the noise severity increased at each step. As the level of corruption increased, a larger portion of noise-augmented training samples was injected into the incremental learning phase. This proportional adjustment of noise–dataset ratio prevented catastrophic forgetting by ensuring that the model continuously retained exposure to clean samples while adapting to gradually intensified noise.

While masking-based partial updates significantly reduce communication bandwidth, they may cause minor accuracy degradation due to the exclusion of low-importance parameters. To evaluate efficiency, the accuracy gain per transmitted megabyte (AccGain/MB) was measured as a combined indicator of adaptation quality and communication cost. A higher value reflects more effective utilization of limited bandwidth and stronger runtime adaptability under edge constraints.

As shown in Figure 7, the proposed masking-based FPGA update achieved 3.5× higher AccGain/MB compared with the GPU full-model update baseline and 1.8× higher than quantized fixed-ratio updates. Although the absolute accuracy of the full retraining baseline was slightly higher within 1.0% difference, the proposed method attained a comparable recovery level while transmitting only 27% of the total model parameters. This result confirms that transmitting only representative parameters can achieve near-optimal adaptation performance while substantially reducing bandwidth usage.

The sparsity of the transmitted mask was varied between 10% and 50%, and the highest AccGain/MB was observed near 25% sparsity, indicating an optimal trade-off between adaptation accuracy and communication cost. Overall, the results validate that the proposed masking-based selective update strategy maximizes adaptation efficiency and ensures real-time responsiveness in bandwidth-constrained edge-cloud systems.

Figure 7g shows the accuracy measured on both the original clean test set and the newly generated noise test set at each adaptation step. The evaluation demonstrates that both the full retraining baseline and the proposed mask-based update method successfully maintain accuracy on the clean dataset, indicating minimal forgetting of previously learned knowledge. At the same time, performance on the noisy dataset consistently improves with each adaptation iteration, confirming that the model gains robustness against the evolving noise distribution.

The results verify that the proposed selective-update approach enables stable multi-step adaptation without accuracy collapse. By combining partial retraining with importance-driven masking, the system simultaneously preserves prior knowledge and enhances performance on novel, noise-corrupted inputs. This confirms the effectiveness of the proposed architecture in long-term, sensor-driven adaptation scenarios where environmental distortions gradually intensify.

## 5. Conclusions

This paper presented a runtime robust edge inference system that leverages FPGA-based DPR and masking-based selective updates to achieve low-latency adaptation under changing environments. The proposed architecture integrates three major modules: an importance analyzer for identifying sensitive parameters through local gradient computation, a partial weight update engine that performs selective retraining for high-importance weights, and an adaptive mask generator that minimizes communication bandwidth by transmitting only representative parameter deltas. By combining these modules with event driven scheduling and FPGA reconfigurability, the system supports real time adaptation while maintaining continuous inference operation.

Experimental results demonstrated that the FPGA implementation on the Alveo U200 achieved up to 1.3 times faster end-to-end adaptation latency compared with GPU-based training. The dynamic partial reconfiguration process required only 0.11–0.17 s per module swap, and the masking based communication path reduced transmitted payloads to less than 30% of the full model size, with only 1.2% accuracy degradation. The proposed architecture thus offers a highly communication-efficient and hardware-adaptive solution for cloud-assisted edge AI systems.

The main advantage of the proposed system lies in its ability to dynamically adjust computation and communication according to runtime conditions. Instead of static deployment, the FPGA server flexibly reconfigures its hardware modules based on scenario-specific needs such as noise adaptation, class extension, or precision control. This enables edge devices to maintain inference robustness with minimal bandwidth consumption and reduced energy cost.

Future work will focus on extending the DPR granularity toward finer layer partitions and supporting multi-task learning with concurrent partial updates. Additionally, integrating the adaptive update pipeline with lightweight edge accelerators and high-speed interconnects is expected to further improve scalability and responsiveness. Overall, the proposed FPGA DPR-based framework provides a promising foundation for developing adaptive, communication-efficient, and hardware reconfigurable edge-cloud AI systems.

## Data Availability

The original contributions presented in the study are included in the article, further inquiries can be directed to the corresponding author.
